# Risk Prediction for Gastric Cancer Using GWAS-Identifie Polymorphisms, *Helicobacter pylori* Infection and Lifestyle-Related Risk Factors in a Japanese Population

**DOI:** 10.3390/cancers13215525

**Published:** 2021-11-03

**Authors:** Naoyo Ishikura, Hidemi Ito, Isao Oze, Yuriko N. Koyanagi, Yumiko Kasugai, Yukari Taniyama, Yukino Kawakatsu, Tsutomu Tanaka, Seiji Ito, Masahiro Tajika, Yasuhiro Shimizu, Yasumasa Niwa, Keitaro Matsuo

**Affiliations:** 1 Division of Cancer Epidemiology and Prevention, Aichi Cancer Center Research Institute, Nagoya 464-8681, Japan; n.ishikura@aichi-cc.jp (N.I.); i_oze@aichi-cc.jp (I.O.); ymaeda@aichi-cc.jp (Y.K.); y.kawakatsu@aichi-cc.jp (Y.K.); kmatsuo@aichi-cc.jp (K.M.); 2 Department of Pathology, International Goodwill Hospital, Yokohama 245-0006, Japan; 3 Division of Cancer Information and Control, Aichi Cancer Center Research Institute, Nagoya 464-8681, Japan; ykoyanagi@aichi-cc.jp (Y.N.K.); y.taniyama@aichi-cc.jp (Y.T.); 4 Division of Descriptive Cancer Epidemiology, Nagoya University Graduate School of Medicine, Nagoya 466-0065, Japan; 5 Department of Endoscopy, Aichi Cancer Center Hospital, Nagoya 464-8681, Japan; tstanaka@aichi-cc.jp (T.T.); mtajika@aichi-cc.jp (M.T.); yshimizu@aichi-cc.jp (Y.S.); 6 Department of Gastrointestinal Surgery, Aichi Cancer Center Hospital, Nagoya 464-8681, Japan; seito@aichi-cc.jp; 7 Aichi Cancer Center Hospital, Nagoya 464-8681, Japan; yniwa@aichi-cc.jp; 8 Division of Cancer Epidemiology, Nagoya University Graduate School of Medicine, Nagoya 466-0065, Japan

**Keywords:** gastric cancer, risk prediction, genetic variants, lifestyle factors, *Helicobacter pylori* infection

## Abstract

**Simple Summary:**

Gastric cancer remains the major cancer in Japan and worldwide. It is expected that practical intervention strategies for prevention, such as personalized approaches based on genetic risk models, will be developed. Here, we developed and validated a risk prediction model for gastric cancer using genetic, biological, and lifestyle-related risk factors. Results showed that the combination of selected GWAS-identified SNP polymorphisms and other predictors provided high discriminatory accuracy and good calibration in both the derivation and validation studies; however, the contribution of genetic factors to risk prediction was limited. The greatest contributor to risk prediction was ABCD classification (*Helicobacter pylori* infection-related factor).

**Abstract:**

Background: As part of our efforts to develop practical intervention applications for cancer prevention, we investigated a risk prediction model for gastric cancer based on genetic, biological, and lifestyle-related risk factors. Methods: We conducted two independent age- and sex-matched case–control studies, the first for model derivation (696 cases and 1392 controls) and the second (795 and 795) for external validation. Using the derivation study data, we developed a prediction model by fitting a conditional logistic regression model using the predictors age, ABCD classification defined by *H. pylori* infection and gastric atrophy, smoking, alcohol consumption, fruit and vegetable intake, and 3 GWAS-identified polymorphisms. Performance was assessed with regard to discrimination (area under the curve (AUC)) and calibration (calibration plots and Hosmer–Lemeshow test). Results: A combination of selected GWAS-identified polymorphisms and the other predictors provided high discriminatory accuracy and good calibration in both the derivation and validation studies, with AUCs of 0.77 (95% confidence intervals: 0.75–0.79) and 0.78 (0.77–0.81), respectively. The calibration plots of both studies stayed close to the ideal calibration line. In the validation study, the environmental model (nongenetic model) was significantly more discriminative than the inclusive model, with an AUC value of 0.80 (0.77–0.82). Conclusion: The contribution of genetic factors to risk prediction was limited, and the ABCD classification (*H. pylori* infection-related factor) contributes most to risk prediction of gastric cancer.

## 1. Introduction

Gastric cancer is the second most common cancer [1] and is the third leading cause of cancer death in men and women [2] in Japan. Despite dramatic declines in incidence and mortality rates in the last several decades, it still confirms its status as a major public health issue in this country. Epidemiological evidence for the development of gastric cancer has been accumulating, and *Helicobacter pylori* (*H. pylori*) infection is now confirmed to be a convincing risk factor for gastric cancer in Japanese [3,4], in addition to the subsequent chronic atrophic gastritis that follows *H. pylori* infection [5]. Stratification by a combination of *H. pylori* infection and atrophic gastritis, known as ABCD classification, was associated with gastric cancer risk in case–control studies [4,6] and well predicted the incidence of gastric cancer in prospective studies [7,8,9,10,11,12,13,14]. In contrast, consumption of fruits and vegetables is recognized as a protective factor in gastric cancer. A meta-analysis of global data showed that fruit and vegetable consumption is associated with a significant reduction in gastric cancer risk [15]. With regard to tobacco, an association with tobacco smoking has been clearly established worldwide [16], including Japan [17], and 11% of gastric cancer cases may be attributed to it [16]. Similarly, alcohol drinking is recognized as a cause of gastric cancer. A large pooled analysis found an association between heavy alcohol drinking and risk of gastric cancer [18].

Recently, genome-wide association studies (GWASs) have enabled genetic discoveries in various types of cancer, including gastric cancer. For example, a single-nucleotide polymorphism (SNP), prostate stem cell antigen (PSCA)-rs2294008, was found to confer susceptibility to gastric cancer risk both in Japan [19,20] and worldwide [21,22,23,24]. This effect was confirmed in a meta-analysis [25]. In addition, GWASs have identified a number of loci that confer susceptibility to gastric cancer, including mucin 1 (MUC1)-rs4072037 [26,27], phospholipase C epsilon 1 (PLCE1)-rs2274223 [27], protein kinase AMP-activated alpha 1 catalytic subunit (PRKAA1)-rs10074991 [26], PRKAA1-rs13361707 [28], Unc-5 family C-terminal like (UNC5CL)-rs2294693 [26], leucine-rich repeat and fibronectin type-III domain containing 2 (LRFN2)-rs2494938 [29], dynein axonemal heavy chain 11 (DNAH11)-rs2285947 [29], zinc finger and BTB domain-containing 20 (ZBTB20)-rs9841504 [28], ASH1-like histone lysine methyltransferase (ASH1L)-rs80142782 [30], LINC02161-rs7712641 [30], ABO, alpha 1-3-N-acetylgalactosaminyltransferase and alpha 1-3-galactosyltransferase (ABO)-rs7849280 [31], cut-like homeobox 2 (CUX2)-rs6490061 [31], and defensin beta 121 (DEFB121)-rs2376549 [31]. These advances in molecular epidemiological findings have the potential to impact cancer prevention. To our knowledge, however, their contribution to the prevention of gastric cancer at the population level in combination with environmental factors has not been evaluated.

In this study, we examined a risk prediction model using these GWAS-identified SNPs and several risk factors of gastric cancer for possible use in distinguishing people at high and low risk of gastric cancer in personalized prevention settings.

## 2. Materials and Methods

### 2.1. Study Population

Two independent case–control studies were conducted to develop a risk prediction model. The study subjects were selected from the participants of the Hospital Epidemiology Research Program at Aichi Cancer Center (HERPACC)-2 (2001–2005) for the derivation study and HERPACC-3 (2005–2013) for the validation study. The frameworks of HERPACC-2 and HERPACC-3 have been described elsewhere [32,33,34]. Briefly, all first-visit outpatients aged 20–79 were recruited to participate in the HERPACC-2 and -3. They were asked to fill in a questionnaire on lifestyle information before their first medical examination and provide blood samples. Response rate for enrollment was 97% for subjects in HERPACC-2, of whom half provided blood samples. In HERPACC-3, 66.4% of participants responded to the questionnaire, of whom 62% provided blood samples. In each study, cases were histologically diagnosed with gastric cancer, and controls were confirmed to have no cancer and no history of neoplasm. Controls were randomly selected and individually matched by age (± 5 years) and sex at a case–control ratio of 1:2 in the derivation study and 1:1 in the validation study. As a result, the present analysis included 696 cases/1392 controls in the derivation study and 795 cases/795 controls in the validation study. Written informed consent was obtained from all participants. The study was approved by the institutional ethics committee of Aichi Cancer Center.

### 2.2. Assessment of Helicobacter pylori Infection and Gastric Atrophy

All cases were examined for plasma IgG level for *H. pylori* using a commercially available direct enzyme-linked immunosorbent assay kit (‘E Plate “Eiken” *H. pylori* Antibody’; Eiken Kagaku, Tokyo, Japan). This kit is commonly used in medical studies in Japan [4,35]. A positive status for *H. pylori* infection was defined as an anti-*H. pylori* IgG antibody level > 10 U/mL in serum [4,35]. Serum pepsinogens (PG) were measured by chemiluminescence enzyme immunoassay, and gastric mucosal atrophy was defined by a PG I value ≤ 70 ng/mL and PG I/PG II ≤ 3 ng/mL [36,37]. We applied the ABCD classification [38,39], using the combination of statuses of *H. pylori* infection (Hp) and gastric atrophy (GA) defined by pepsinogen levels. Participants were classified into four groups: Group A [Hp(−) GA(−)], Group B [Hp(+) GA(−)], Group C [Hp(+) GA(+)], and Group D [Hp(−) GA(+)]).

### 2.3. Information on Lifestyle Risk Factors

To select lifestyle factors, we referred to Development and Evaluation of Cancer Prevention Strategies in Japan [3] and extracted risk/preventive factors for gastric cancer. In this matrix, smoking and *H. pylori* infection are certain risk factors, and vegetable and fruit intake are possible preventative factors for both men and women. Cereal intake (possible risk factor) and salt intake (almost certain risk factor) are omitted from lifestyle risk factors, as they cannot be estimated by our food frequency questionnaire.

Information on lifestyle factors was collected by a self-administered questionnaire. Smoking status was classified into three categories of never smoker, former smoker, and current smoker, with former smokers defined as those who had quit at least 1 year before study enrolment. Alcohol consumption status was classified into four categories: never, low, moderate, and heavy. Those who seldom or never drank were defined as never drinkers. Low drinking was defined as consumption on 4 days or fewer per week, moderate drinking as consumption of less than 46 g of ethanol on 5 days or more per week, and heavy drinking as consumption of more than 46 g ethanol on 5 days or more per week. Information on family history of gastric cancer was obtained in the two categories of yes and no regarding a history of gastric cancer in any first-degree relative. Consumption of fruits and vegetables was determined using a food frequency questionnaire, which included 43 single food items in eight frequency categories [40]. The food frequency questionnaire was validated using a 3-day weighed dietary record as standard, which showed that reproducibility and validity were satisfactory [40,41]. Participants were divided into three groups based on the distribution of fruit and vegetable consumption among controls in the derivation study (tertiles).

### 2.4. Examination of the GWAS-Identified Polymorphisms

We conducted literature searches through PubMed (https://www.ncbi.nlm.nih.gov/pubmed) on 1 November 2017, to select GWAS-identified polymorphisms for evaluation in this study. We selected fourteen gastric cancer-susceptible SNPs reported in previous GWASs, namely PSCA-rs2294008 [21], MUC1-rs4072037 [27], PLCE1-rs2274223 [27], PRKAA1-rs10074991 [26], PRKAA1-rs13361707 [28], UNC5CL-rs2294693 [26], LRFN2-rs2494938 [29], DNAH11-rs2285947 [29], ZBTB20-rs9841504 [28], ASH1L-rs80142782 [30], LINC02161-rs7712641 [30], ABO-rs7849280 [31], CUX2-rs6490061 [31], and DEFB121-rs2376549 [31], as candidate genetic factors for risk prediction. DNA in the buffy coat fraction of each participant was extracted using a QIAmp DNA blood mini kit (Qiagen K.K., Tokyo, Japan). The selected SNPs were genotyped using TaqMan Single Nucleotide Polymorphism Genotyping Assays (Applied Biosystems, Foster City, CA, USA). The quality of genotyping in our laboratory is routinely assessed by statistical evaluation using the Hardy–Weinberg test and re-genotyping of 5% of randomly sampled subjects.

### 2.5. Statistical Analysis

To create a risk prediction model, we selected established environmental and lifestyle factors of gastric cancer (smoking (never, former and current), alcohol consumption (never, moderate, high–moderate, and heavy), energy-adjusted fruit and vegetable intake (in tertiles among controls in the derivation study), family history of gastric cancer (first-degree relative), and ABCD classification (in indicator variables, A, B, C, and D)). We examined the impact of each risk factor by conditional logistic regression. Age as continuous, sex, family history of gastric cancer, and referral pattern were included as adjusted factors in the model. Subjects with an unknown status for these variables were assigned dummy variables for the missing categories and included in the analysis. To assess the specific impact of a selected factor, we estimated the odds ratios (ORs) and corresponding 95% confidence intervals (CI) using uni- and multivariable conditional logistic regression models in the derivation study. For genetic factors, we evaluated the impact of each polymorphism by OR, 95% CI, and *p*-value adjusted for age and sex in both studies. These were calculated using the per-allele model of conditional logistic regression. To create risk prediction models, we selected polymorphisms with a value of *p* < 0.01 in the derivation study as risk predictors.

Performance of the risk prediction model was assessed in both the derivation study (as “internal validation”) and the validation study (as “external validation”) using standard methods for measurement of discrimination and calibration [42]. Discriminability was assessed by calculating the area under the curve (AUC) in the receiver-operating characteristic (ROC) curve, commonly known as the concordance (c) statistic. In the ROC, sensitivity is shown on the *y*-axis and false positive rate on the *x*-axis; a straight line in ROC indicates random classification of cases and controls, with a minimum AUC of 0.5. An AUC value of 1 corresponds to perfect classification, while values of 0.7 and 0.8 rate the model as having acceptable discrimination ability and above 0.8 as having excellent discrimination ability [43]. The AUC values were compared using the method of DeLong et al. [44]. The calibration of the models was assessed by the Hosmer–Lemeshow goodness-of-fit statistic and calibration plots. Subjects were divided into subgroups by decile of predicted probability. The Hosmer–Lemeshow statistic is computed based on a χ2-test, which compares the observed frequencies with the predicted frequencies in the ten groups; a nonsignificant *p*-value indicates good calibration, whereas a significant *p*-value indicates disagreement between the predicted and observed outcomes. In a calibration plot, the mean predicted probability was plotted against the mean observed probability for each decile. Ideally, the predicted probability equals the observed probability, so perfect predictions should lie on the 45° line [42]. In addition, with perfect calibration, the estimated calibration slope equals 1 [45]. A slope below 1.0 reflects overfitting of the model [46], which indicates the need to shrink the regression coefficients [42].

All analyses were performed using Stata/SE 14 (Stata Corp, College Station, TX, USA).

## 3. Results

The two case–control studies were largely comparable (Table 1). The proportion of current smokers was higher in cases than controls in both (42.2% and 30.9% in the derivation study and 31.7% and 22.8% in the validation study, respectively), as was the prevalence of *H. pylori* infection (82.2% and 55.6 in the derivation study and 71.7% and 41.9% in the validation study, respectively). Cases were more likely to have daily fruit and vegetable consumption than controls in both studies. Alcohol consumption and family history showed no apparent difference between cases and controls.

Table 2 shows associations of selected lifestyle-related or biological factors in our prediction model, namely smoking, alcohol consumption, fruit and vegetable intake, and the ABCD classification with gastric cancer risk. We observed a statistically significant association with each selected factor in both studies, with the exception of alcohol consumption and fruit and vegetable intake. The results of the validation study and meta-analysis are presented in Table S1.

Table 3 presents the association between 14 polymorphisms and gastric cancer risk. We selected three polymorphisms, namely rs4072037, rs2294008 and rs7849280, with values of *p* < 0.01, to develop a risk prediction model. The results of the validation study and meta-analysis are presented in Table S2.

**Figure 1:**
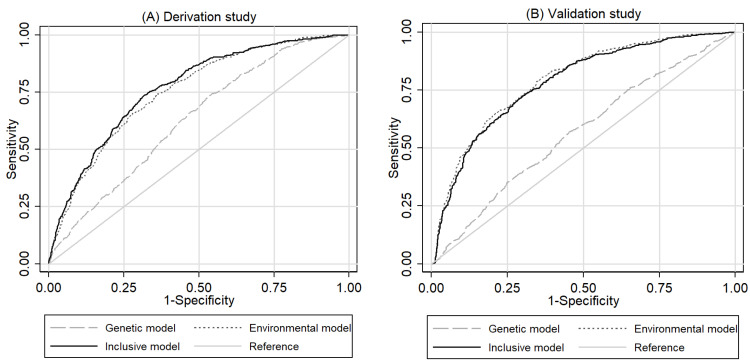
Area under the curve (AUC) of the derivation (**A**) and validation (**B**) studies. In the derivation study, the inclusive model had a statistically significantly higher discriminatory ability than the other models. In the validation study, however, the environmental model was significantly more discriminative than the inclusive model.

**Figure 2:**
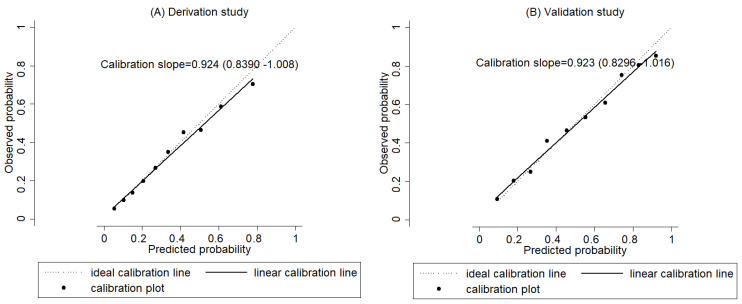
Calibration slope in the derivation (**A**) and validation (**B**) study (inclusive model). The calibration plots of the inclusive model stayed close to the ideal calibration line throughout the risk spectrum in all data sets of both studies, and calibration slopes were close to 1.0.

Next, we assessed the performance of the prediction model (Table 4 and Table 5; Figure 1 and Figure 2). The discriminative abilities in the validation study were similar to those in the derivation study. The inclusive model provided acceptable discrimination in both the derivation and validation studies with AUC values of 0.7677 (0.7465–0.789) and 0.7823 (0.7694 chromosome 0.814), respectively (Table 4 and Figure 1). In the derivation study, the inclusive model had a statistically significantly higher discriminatory ability than the other genetic and nongenetic models (*p* = 4.74 × 10^−53^). In the validation study, however, the environmental model was significantly more discriminative than the inclusive model, with an AUC value of 0.7925 (0.7705–0.815). The calibration analysis of the inclusive model revealed reasonably good agreement between the observed and predicted number of gastric cancer cases in groups defined by deciles of predicted risk distribution in both the derivation (*p* for Hosmer–Lemeshow test = 0.445) and validation studies (*p* = 0.116) (Table 5). Moreover, the calibration plots of the inclusive model stayed close to the ideal calibration line throughout the risk spectrum in all data sets of both studies (Figure 2), and all of their calibration slopes were close to 1.0.

## 4. Discussion

In this study, we developed a risk prediction model of gastric cancer using a combination of genetic, biological, and lifestyle-related risk factors. In the derivation study, discriminatory ability was slightly improved in the inclusive model, which consisted of both genetic and biological and lifestyle-related factors, than in the models that included only biological and lifestyle-related risk factors (environmental model). In the validation study, however, the environmental model was more discriminating than the inclusive model. The addition of genetic factors (SNPs) improved the performance of the risk prediction model only slightly, which suggests that genetic factors are less useful for risk prediction.

This study represents the first attempt to combine genetic, biological, and lifestyle-related risk factors in the prediction of gastric cancer risk. Several previous risk prediction models for gastric cancer were investigated in large-scale population-based cohort studies in Japan, but these did not include genetic factors. Namely, Charvat et al. developed a prediction model to estimate an individual’s risk of gastric cancer in Japan using a combination of age, sex, smoking, salted food consumption, family history of gastric cancer, and the ABCD classification [12], while Iida et al. developed a model in a cohort study in Japan using a combination of age, sex, combination of anti-*H. pylori* antibody and atrophic gastritis, hemoglobin A1c, smoking, drinking, and obesity [13]. In addition, Cai et al. recently developed a gastric cancer risk prediction rule in China based on a combination of age, sex, PG I/II ratio, gastrin-17 level, *H. pylori* infection, pickled food, and fried food. These models showed good performance, but did not include genetic factors [47].

Here, we selected MUC1-rs4072037, PSCA-rs2294008, and ABO-rs7849280 as genetic risk factors. MUC1-rs4072037 was identified in GWASs [26,27] and replicated in case–control studies [30,48] in East Asian countries. The membrane mucin MUC1 is a ligand for *H. pylori* in the stomach, and the SNP rs4072037 is known to determine a splicing acceptor site in the second exon of MUC1 [49]. MUC1-rs4072037 is an independent risk factor that influences tumor recurrence and disease-related death in diffuse-type gastric cancer, but not in intestinal-type gastric cancer [48]. PSCA-rs2294008 is a GWAS-identified susceptibility polymorphism for gastric cancer both in Japan and worldwide [19,20,21,22,24,25,50]. PSCA is expressed in differentiating gastric epithelial cells, shows a cell proliferation inhibitory effect in vitro, and is frequently downregulated in gastric cancer. PSCA-rs2294008 is a functional SNP that influences the transcriptional activity of the PSCA promoter; the T allele significantly suppresses its transcription activity, thus affecting susceptibility to diffuse-type gastric cancer [21]. ABO-rs7849280 was identified in a Japanese GWAS [31]. An association between blood type A and gastric cancer has been previously reported [51,52]. Tanikawa et al. revealed that the AA blood type has a higher frequency of the G allele of ABO-rs7849280 than other types. The risk G allele was associated with higher ABO mRNA expression, whereas ABO mRNA expression was significantly suppressed in *H. pylori*-infected stomach [31]. ABO-rs7849280 is a key regulator of host-bacterial interactions of *H. pylori*-related diseases and gastric cancer.

Among environmental and biological factors, the ABCD classification showed a particularly high AUC value. Consistent with previous studies, we found that *H. pylori* infection and gastric atrophy substantially impacted gastric cancer risk. Their contribution to risk prediction was considerable, with AUCs 0.7354 and 0.7885 in the ABCD classification in the derivation and validation studies, respectively. Group D is negative for *H. pylori* infection but confers a high risk of gastric cancer. It is well known that *H. pylori* can no longer survive when atrophy has severely progressed or in the metaplastic intestinal mucosa induced by *H. pylori* infection [13], and production of anti-*H. pylori* antibodies in these conditions may be reduced. Therefore, although the subjects in Group D were not positive for *H. pylori* infection, most had been previously infected and were therefore also at high risk of developing gastric cancer, such as those in Group C.

The addition of novel GWAS-identified susceptibility loci may contribute to improving the performance of risk models. To date, however, the degree of such improvement has remained unclear. For example, in Szulkin et al.’s prostate cancer risk prediction study [53], a polygenic risk score for 65 established susceptibility variants provided an area under the curve (AUC) of 0.67, and the addition of 68 new variants increased the AUC to 0.68. In a similar study of the development of polygenic risk scores for prediction of breast cancer [54], the AUC of the prospective study was 0.603 with 77 SNPs, 0.630 with 313 SNPs, and 0.636 with 3820 SNPs. These findings suggest that the addition of SNPs may improve performance, albeit only to a limited degree.

In our present study, we also investigated the effects of increasing the number of SNPs. We selected SNPs with values of *p* < 0.05 in the derivation study (rs2294008, rs4072037, rs7849280, rs10074991, rs2294693, rs80142782) and then used the six SNPs selected to construct a new genetic model. As a supplementary explanation, rs13361707 also has a value of *p* < 0.05. Since both rs10074991 and rs13361707 are SNPs for PRKAA1 and in linkage disequilibrium, we chose rs10074991, which has a smaller *p*-value. Results showed an improvement in the AUC of the inclusive model in both the derivation (0.7728) and validation studies (0.7871). However, even with the inclusion of these six SNPs, the AUC of the environmental model was higher than that of the inclusive model in the validation study. In addition, in calibration using the Hosmer–Lemeshow test, *p* = 0.018 in the validation study, and the calibration could not be performed. Accordingly, although the addition of genetic factors had a positive effect on improving validation ability, these were not as great as expected due to the large impact of *H. pylori* infection and ABCD classification. For cancers that are significantly affected by one environmental factor, such as *H. pylori* in gastric cancer, the contribution of genetic risk factors to risk prediction may be limited.

Our study has several strengths. First, it was relatively large, and information was available on genetics, as well as *H. pylori* infection status, serologically defined gastric atrophy, and lifestyle characteristics. This allowed us to provide reliable estimates of risk factor effects and the performance of the model. Second, the constructed model was validated in a different dataset. Third, potential confounding by age and sex were considered by matching. Fourth, the allele frequencies of each SNP in the controls of this study were similar to that reported in HapMap JPT (available at http://www.ncbi.nlm.nih.gov/snp, accessed on 10 June 2020), warranting the comparability of our results for genetic factors with those in general populations in Japan.

Several limitations should also be noted. First, our lifestyle factors were obtained in a retrospective manner, and *H. pylori*/atrophy information was obtained in a cross-sectional setting. Validation of the model in prospective studies is clearly warranted, and until then, application in prospective settings requires caution. Second, *H. pylori* infection and gastric mucosal atrophy status were defined by serological tests. Cutoff levels for defining negativity of serum anti-*H. pylori* antibody titers are reported to be too high [55], and *H. pylori* infection status might have been wrongly classified. If so, this might have introduced status misclassification, which would nevertheless have been nondifferential. Accordingly, the impact of these factors may have been underestimated. Third, although salt intake is known as a “probable” risk factor for gastric cancer [3] and the attributable fraction of salt intake is not negligible [56], it was not considered in the study. The addition of salt information might improve model performance, and should therefore be considered for future studies. Finally, residual confounding by known and unknown factors in the model might be present.

## 5. Conclusions

We developed and validated a risk prediction model for gastric cancer using genetic, biological, and lifestyle-related factors in Japanese. The contribution of genetic factors to risk prediction was limited due to the large impact of the ABCD classification (*H. pylori* infection-related factor).

## Figures and Tables

**Table 1:** Characteristics of participants.

Characteristics		Derivation Study				Validation Study			
		Cases		Controls		Cases		Controls	
		*n* = 696	33.33%	*n* = 1392	66.67%	*n* = 795	50.00%	*n* = 795	50.00%
Sex	Male	520	74.7	1040	74.7	590	74.2	590	74.2
	Female	176	25.3	352	25.3	205	25.8	205	25.8
Age (years)	<40	34	4.9	74	5.3	34	4.3	35	4.4
	40–49	72	10.3	174	12.5	62	7.8	77	9.7
	50–59	244	35.1	436	31.3	212	26.7	201	25.3
	60–69	210	30.2	458	32.9	319	40.1	310	39.0
	>70	136	19.5	250	18.0	168	21.1	172	21.6
Smoking status	Never	222	31.9	538	38.7	277	34.8	321	40.4
	Former	180	25.9	423	30.4	263	33.1	292	36.7
	Current	294	42.2	430	30.9	252	31.7	181	22.8
	Unknown	0	0.0	1	0.1	3	0.4	1	0.1
Alcohol consumption	Never	228	32.8	469	33.7	284	35.7	268	33.7
	Low	250	35.9	558	40.1	253	31.8	309	38.9
	Moderate	113	16.2	209	15.0	122	15.4	114	14.3
	Heavy	86	12.4	130	9.3	130	16.4	101	12.7
	Unknown	19	2.7	26	1.9	6	0.8	3	0.4
Fruit/vegetable intake ^a^	High intake	259	37.2	448	32.2	317	39.9	278	35.0
	Moderate intake	215	30.9	477	34.3	251	31.6	249	31.3
	Low intake	220	31.6	458	32.9	227	28.6	268	33.7
	Unknown	2	0.3	9	0.7	0	0.0	0	0.0
Family history of gastric cancer	Yes	153	22.0	244	17.5	196	24.7	136	17.1
	No	543	78.0	1148	82.5	599	75.4	659	82.9
*H.pylori* (Hp) IgG test	Negative	124	17.8	618	44.4	225	28.3	462	58.1
	Positive	572	82.2	774	55.6	570	71.7	333	41.9
Gastric atrophy (GA) defined by PG testing	Negative	261	37.5	910	65.4	426	53.6	637	80.1
	Positive	435	62.5	482	34.6	369	46.4	158	19.9
	Unknown	0	0.0	0	0.0				
ABCD classification	Group A [Hp(−) GA(−)]	68	9.77	558	40.1	150	18.9	446	56.1
	Group B [Hp(+) GA(−)]	193	27.73	352	25.3	276	34.7	191	24.0
	Group C [Hp(+) GA(+)]	379	54.45	422	30.3	294	37.0	142	17.9
	Group D [Hp(−) GA(+)]	56	8.05	60	4.3	75	9.4	16	2.0
Referral pattern to hospital	Patient’s discretion	126	18.1	423	30.39	112	14.09	126	15.85
	Family recommendation	119	17.1	229	16.45	135	16.98	66	8.3
	Referral from another clinic	308	44.25	367	26.36	294	36.98	250	31.45
	Secondary screening after primary screening	135	19.4	333	23.92	187	23.5	269	33.84
	Other	3	0.43	8	0.57	4	0.5	7	0.88
	Unknown	5	0.72	32	2.3	63	7.92	77	9.69

^a^ Third quantile of fruits/vegetables is the third quantile of intake in the control group. As tertiles for HERPACC2 and HERPACC3 were calculated separately, cutoff values differ between the derivation study (HERPACC2) and validation study (HERPACC3), as follows. Derivation study: high intake (197.43 g/day or more), moderate intake (109.35–197.43 g/day), and low intake (less than 109.35 g/day). Validation study: high intake (197.59 g/day or more), moderate intake (109.41–197.59 g/day), and low intake (less than 109.41 g/day).

**Table 2:** Associations of epidemiological and clinical risk factors in stomach cancer (derivation study).

Characteristics		Derivation Study					
		Model 1			Model 2		
		OR ^a^ (95% CI)			OR ^b^ (95% CI)		
Smoking ^c^							
	Never	Reference					
	Former	1.16	(0.88–1.53)		1.07	(0.78–1.47)	
	Current	1.89	(1.47–2.44)		1.81	(1.34–2.46)	
	*p* for trend	1.75 × 10^−7^			2.59 × 10^−5^		
Alcohol consumption ^c^							
	Never	Reference			Reference		
	Low	0.94	(0.74–1.18)		0.93	(0.72–1.21)	
	Moderate	1.12	(0.84–1.50)		1.01	(0.72–1.41)	
	Heavy	1.40	(1.00–1.96)		1.29	(0.86–1.94)	
	*p* for trend	3.38 × 10^−2^			2.30 × 10^−1^		
Fruit and vegetable intake ^c^							
	Highest tertile	Reference			Reference		
	Middle tertile	0.95	(0.76–1.20)		0.88	(0.67–1.14)	
	Lowest tertile	1.22	(0.96–1.54)		1.01	(0.77–1.33)	
	*p* for trend	9.32 × 10^−2^			9.05 × 10^−1^		
*H. pylori* infection (Hp)							
	Negative	Reference			Reference		
	Positive	3.57	(2.84–4.47)		2.54	(1.96–3.28)	
	*p* for trend	3.57 × 10^−28^			1.08 × 10^−12^		
Gastric atrophy (GA) ^c^							
	Negative	Reference			Reference		
	Positive	3.29	(2.69–4.03)		2.54	(2.01–3.20)	
	*p* for trend	8.22 × 10^−31^			4.00 × 10^−15^		
ABCD stratification ^c^							
(Hp and GA)	A: Hp-Negative; GA-Negative	Reference			Reference		
	B: Hp-Positive; GA-Negative	4.45	(3.23–6.12)		4.42	(3.15–6.18)	
	C: Hp-Positive; GA-Positive	7.67	(5.64–10.43)		8.09	(5.81–11.25)	
	D: Hp-Negative; GA-Positive	8.28	(5.21–13.17)		8.84	(5.38–14.53)	
	*p* for trend	1.21 × 10^−38^			4.27 × 10^−36^		

OR, odds ratio; CI, confidence interval. ^a^ Crude OR by the conditional logistic regression model. For Fruit and vegetable intake, ORs were adjusted by energy intake. ^b^ For smoking and fruit and vegetable intake, odds ratios were calculated by a conditional logistic regression model adjusted for age at first visit and family history of gastric cancer, smoking status, drinking habit (alcohol drinking (ethanol (g/day)), energy-adjusted fruit and vegetable intake, energy intake, *H. pylori* infection, gastritis atrophy, and referral pattern. For *H. pylori* infection, gastric atrophy, and ABCD classification, odds ratios were calculated by a conditional logistic regression model adjusted for age at first visit and family history of gastric cancer, smoking status, drinking habit, energy-adjusted fruit and vegetable intake, energy intake, and referral pattern. ^c^ Subjects with unknown status are excluded from each analysis.

**Table 3:** Associations with Asian GWAS-identified susceptibility polymorphism in stomach cancer risk (derivation study).

Reference	Chromosome	Position	Genes in/near Region	SNP	Genotype Risk/Non-Risk Alleles	Derivation Study (Case/Control = 696/1372)			
						Risk Allele Frequency in Controls	OR ^a^ (95%CI), per Allele		
							*p*		
Abnet CC, et al. [27]	1q22	155192276	MUC1	rs4072037	G/A	0.824	1.35	(1.13–1.61)	
							1.07 × 10^−3^		
Abnet CC, et al. [27]	10q23	94306584	PLCE1	rs2274223	G/A	0.744	1.09	(0.94–1.26)	
							2.62 × 10^−1^		
Hu N, et al. [26]	5p13.1	40790449	PRKAA1	rs10074991	G/A	0.444	1.18	(1.04–1.35)	
							1.34 × 10^−2^		
Hu N, et al. [26]	6p21.1	41037763	UNC5CL	rs2294693	C/T	0.234	1.16	(1.00–1.35)	
							5.19 × 10^−2^		
Jin G et al. [29]	6p21.1	40568389	LRFN2	rs2494938	G/A	0.683	0.98	(0.86–1.13)	
							8.02 × 10^−1^		
Jin G et al. [29]	7p15.3	21544470	DNAH11	rs2285947	G/A	0.331	1.09	(0.95–1.25)	
							2.28 × 10^−1^		
Sakamoto H, et al. [21]	8q24.3	142680513	PSCA	rs2294008	T/C	0.619	1.42	(1.23–1.63)	
							1.23 × 10^−6^		
Shi Y, et al. [28]	3q13.31	114643917	ZBTB20	rs9841504	C/G	0.800	1.04	(0.88–1.23)	
							6.67 × 10^−1^		
Shi Y, et al. [28]	5p13.1	40791782	PRKAA1	rs13361707	T/C	0.445	1.18	(1.03–1.35)	
							1.45 × 10^−2^		
Wang Z, et al. [30]	1q22	155515236	ASH1L	rs80142782	T/C	0.965	1.61	(1.07–2.43)	
							2.27 × 10^−2^		
Wang Z, et al. [30]	5q14.3	89607147	NA	rs7712641	T/C	0.388	1.06	(0.93–1.21)	
							3.94 × 10^−1^		
Tanikawa C, et al. [31]	9q34.2	133251249	ABO	rs7849280	G/A	0.235	1.39	(1.20–1.61)	
							1.13 × 10^−5^		
Tanikawa C, et al. [31]	12q24.11-12	111335541	CUX2	rs6490061	C/T	0.683	1.05	(0.91–1.21)	
							4.98 × 10^−1^		
Tanikawa C, et al. [31]	20q11.21	31411284	DEFB121	rs2376549	C/T	0.268	1.12	(0.96–1.29)	
							1.45 × 10^−1^		

OR, odds ratio; CI, confidence interval. ^a^ ORs were adjusted for age and sex.

**Table 4:** Assessment of performance of the prediction model.

Risk Factors	Derivation Study ^b^				Validation Study ^c^			
Area under the curve (95% CI) ^a^				Std. Err.				Std. Err.
Genetic factors								
rs2294008	0.6063	(0.5816–0.631)		0.0126	0.5800	(0.5519–0.608)		0.0143
rs7849280	0.5997	(0.5751–0.624)		0.0125	0.5412	(0.5128–0.570)		0.0145
rs4072037	0.5889	(0.5643–0.614)		0.0126	0.5537	(0.5254–0.582)		0.0144
3 SNPs (rs2294008 rs7849280 rs4072037, Genetic Model)	0.6287	(0.6039–0.653)		0.0126	0.5673	(0.5391–0.596)		0.0144
Environmental Factors								
Smoking	0.6157	(0.5912–0.640)		0.0125	0.5948	(0.5669–0.623)		0.0142
Alcohol consumption	0.5751	(0.5503–0.600)		0.0127	0.5484	(0.5200–0.577)		0.0145
Fruit and vegetable intake	0.5822	(0.5577–0.607)		0.0125	0.5697	(0.5415–0.598)		0.0144
ABCD classification	0.7354	(0.7135–0.757)		0.0112	0.7885	(0.7662–0.811)		0.0114
4 environmental factors (Smoking + Fruit and Vegetable intake + Alcohol Intake + ABCD Classification, Environmental Model)	0.7531	(0.7314–0.775)		0.0111	0.7925	(0.7705–0.815)		0.0112
Inclusive Model (3 SNPs and 4 environmental factors)	0.7677	(0.7465–0.789)		0.0108	0.7823	(0.7694–0.814)		0.0115
*p*-values for comparing genetic, environmental, and inclusive models	4.74 × 10^−53^				5.44 × 10^−64^			
*p*-values for comparing genetic and inclusive models	7.35 × 10^−28^				3.96 × 10^−56^			
*p*-values for comparing environmental and inclusive models	4.64 × 10^−4^				7.17 × 10^−3^			

^a^ Age at first visit is included in each model. ^b^ Range of standard error of AUC is from 0.0108 to 0.0127. ^c^ Range of standard errors of AUC is from 0.0112 to 0.0145.

**Table 5:** Calibration in the deviation and validation study.

Calibration of the Inclusive Model	Derivation Study		Validation Study	
Hosmer–Lemeshow test (hl) (*p*-value)	9.952	(0.445)	15.454	(0.116)
Calibration slope (95%CI)	0.924	(0.8390–1.008)	0.923	(0.8296–1.016)

## Data Availability

The data are not publicly available due to ethical and data security requirements.

## Supplementary Materials

The following are available online at https://www.mdpi.com/article/10.3390/cancers13215525/s1, Table S1: Associations of epidemiological and clinical risk factors in stomach cancer (validation study and meta-analysis), Table S2: Associations with Asian GWAS-identifed susceptibility polymorphism in stomach cancer risk (validation study and meta-analysis).
