# Supplementary material for: Risk Prediction for Gastric Cancer Using GWAS-Identifie Polymorphisms, Helicobacter pylori Infection and Lifestyle-Related Risk Factors in a Japanese Population
Source: Cancers (Basel). 2021 Nov 3;13(21):5525. doi: 10.3390/cancers13215525 (PMC8583059; doi:10.3390/cancers13215525)
Supplement: Supplementary file 1 [file cancers-13-05525-s001.zip › cancers-1384330-supplementary.pdf]

cancers

MDPI

Supplementary Materials

Risk Prediction for Gastric Cancer Using GWAS-identifie Polymorphisms, *Helicobacter pylori* Infection and Lifestyle-Related Risk Factors in a Japanese Population

Naoyo Ishikura, Hidemi Ito, Isao Oze, Yuriko N. Koyanagi, Yumiko Kasugai, Yukari Taniyama, Yukino Kawakatsu, Tsutomu Tanaka, Seiji Ito, Masahiro Tajika, Yasuhiro Shimizu, Yasumasa Niwa and Keitaro Matsuo

Table S1. Associations of epidemiological and clinical risk factors in stomach cancer (validation study and meta-analysis)

Characteristics		Validation study				Meta-analysis			
		Model 1		Model 2		Model 1		Model 2	
		OR[a] (95% CI)		OR[b] (95% CI)		OR[a] (95% CI)		OR[b] (95% CI)	
Smoking [c]									
	Never	Reference		Reference		Reference		Reference	
	Former	1.12	(0.86 -1.47)	1.36	(0.99 -1.87)	1.14	(0.94 -1.38)	1.20	(0.95 -1.52)
	Current	1.79	(1.35 -2.39)	1.70	(1.21 -2.39)	1.85	(1.53 -2.23)	1.76	(1.41 -2.21)
	p for trend	6.44×10^{-5}		0.24×10^{-2}		5.37×10^{-11}		2.49×10^{-7}	
Alcohol consumption [c]									
	Never	Reference		Reference		Reference		Reference	
	Low	0.94	(0.74 -1.18)	0.84	(0.62 -1.13)	0.86	(0.71 -1.03)	0.89	(0.73 -1.08)
	Mod	1.12	(0.84 -1.50)	1.00	(0.69 -1.46)	1.07	(0.87 -1.33)	1.00	(0.78 -1.29)

	Heavy	1.40	(1.00 -1.96)	1.16	(0.77 -1.75)	1.31	(1.04 -1.67)	1.23	(0.92 -1.64)
	p for trend	3.38×10^{-2}		4.67×10^{-1}		1.83×10^{-2}		0.17	
Fruit and vegetable intake [c]									
	Highest tertile	Reference		Reference		Reference		Reference	
	Middle tertile	1.41	(1.08 -1.83)	1.11	(0.81 -1.53)	1.30	(1.09 -1.55)	1.05	(0.85 -1.30)
	Lowest tertile	1.21	(0.94 -1.56)	1.04	(0.76 -1.42)	1.07	(0.84 -1.35)	0.94	(0.77 -1.15)
	p for trend	1.11×10^{-2}		5.14×10^{-1}		8.80×10^{-1}		6.05×10^{-1}	
H. pylori infection (Hp)									
	Negative	Reference		Reference		Reference		Reference	
	Positive	3.47	(2.76 -4.35)	2.69	(2.08 -3.49)	3.52	(3.00 -4.13)	2.61	(2.18 -3.14)
	p for trend	6.83×10^{-27}		6.93×10^{-14}		2.41×10^{-53}		5.25×10^{-25}	
Gastric atrophy (GA) [c]									
	Negative	Reference		Reference		Reference		Reference	
	Positive	3.57	(2.80 -4.56)	2.50	(1.90 -3.28)	3.40	(2.91 -3.98)	2.52	(2.11 -3.01)
	p for trend	2.13×10^{-24}		6.36×10^{-11}		1.93×10^{-53}		1.67×10^{-24}	
ABCD stratification [c]									
(Hp & GA)	Group A [Hp(-) GA(-)]	Reference		Reference		Reference		Reference	
	Group B [Hp(+) GA(-)]	4.50	(3.33 -6.08)	4.36	(3.17 -5.98)	4.48	(3.60 -5.57)	4.38	(3.48 -5.52)
	Group C [Hp(+) GA(+)]	6.36	(4.62 -8.74)	6.27	(4.49 -8.76)	7.00	(5.61 -8.74)	7.13	(5.55 -9.15)
	Group D [Hp(-) GA(+)]	17.96	(9.21 -35.01)	15.62	(7.74 -31.53)	11.73	(5.52 -24.95)	11.10	(6.43 -19.18)
	p for trend	2.85×10^{-37}		1.12×10^{-32}		6.44×10^{-39}		7.43×10^{-67}	

OR, odds ratio; CI, confidence interval. [a] Crude OR by the conditional logistic regression model. For Fruit and vegetable intake, ORs were adjusted by energy intake. [b] For smoking and fruit and vegetable intake, odds ratios were calculated by a conditional logistic regression model adjusted for age at first visit and family history of gastric cancer, smoking status, drinking habit (alcohol drinking (ethanol (g/day)), energy-adjusted fruit and vegetable intake, energy intake, *H. pylori* infection, gastritis atrophy, and referral pattern. For *H. pylori* infection, gastric

atrophy, and ABCD classification, odds ratios were calculated by a conditional logistic regression model adjusted for age at first visit and family history of gastric cancer, smoking status, drinking habit, energy-adjusted fruit and vegetable intake, energy intake, and referral pattern. [c] Subjects with unknown status are excluded from each analysis.

Table S2. Associations with Asian GWAS- identified susceptibility polymorphism in stomach cancer risk (validation study and meta-analysis)

Reference	Chromosome	Position	Genes in/near region	SNP	Genotypes Risk/non-risk alleles	Validation study (case/control=795/795)			Meta-analysis (Derivation and Validation studies)		
						Risk allele frequency in controls	OR [a] (95%CI), per allele *p*		OR [a] (95%CI), per allele *p*		*p* for heterogeneity
Abnet CC, et al [1]	1q22	155192276	MUC1	rs4072037	G/A	0.816	1.12	(0.93 -1.35)	1.23	(1.03 -1.48)	0.157
							2.27×10^{-1}		2.51×10^{-2}		
Abnet CC, et al [1]	10q23	94306584	PLCE1	rs2274223	G/A	0.727	1.11	(0.95 -1.30)	1.10	(0.99 -1.23)	0.83
							1.79×10^{-1}		8.26×10^{-2}		
Hu N, et al. [2]	5p13.1	40790449	PRKAA1	rs10074991	G/A	0.436	1.24	(1.07 -1.43)	1.21	(1.10 -1.33)	0.638
							3.70×10^{-3}		1.55×10^{-4}		
Hu N, et al. [2]	6p21.1	41037763	UNC5CL	rs2294693	C/T	0.252	1.21	(1.03 -1.42)	1.18	(1.06 -1.32)	0.721
							2.32×10^{-2}		3.00×10^{-3}		
Jin G et al. [3]	6p21.1	40568389	LRFN2	rs2494938	G/A	0.677	0.92	(0.79 -1.06)	0.95	(0.86 -1.05)	0.502
							2.48×10^{-1}		3.31×10^{-1}		
Jin G et al. [3]	7p15.3	21544470	DNAH11	rs2285947	G/A	0.340	1.01	(0.87 -1.18)	1.05	(0.95 -1.17)	0.489
							8.74×10^{-1}		3.17×10^{-1}		

Sakamoto H, et al. [4]	8q24.3	142680513	PSCA	rs2294008	T/C	0.608	1.21	(1.04 -1.41)	1.31	(1.13 -1.53)	0.141
							1.16×10^{-2}		4.00×10^{-4}		
Shi Y, et al. [5]	3q13.31	114643917	ZBTB20	rs9841504	C/G	0.800	1.06	(0.89 -1.27)	1.05	(0.93 -1.18)	0.843
							4.96×10^{-1}		4.35×10^{-1}		
Shi Y, et al. [5]	5p13.1	40791782	PRKAA1	rs13361707	T/C	0.438	1.23	(1.07 -1.42)	1.20	(1.09 -1.33)	0.658
							4.47×10^{-3}		1.97×10^{-4}		
Wang Z, et al. [6]	1q22	155515236	ASH1L	rs80142782	T/C	0.963	1.43	(0.95 -2.13)	1.51	(1.14 -2.02)	0.673
							8.39×10^{-2}		4.67×10^{-3}		
Wang Z, et al. [6]	5q14.3	89607147	NA	rs7712641	T/C	0.384	1.07	(0.93 -1.23)	1.07	(0.97 -1.17)	0.907
							3.27×10^{-1}		1.96×10^{-1}		
Tanikawa C, et al [7]	9q34.2	133251249	ABO	rs7849280	G/A	0.262	1.01	(0.87 -1.19)	1.19	(0.87 -1.62)	0.004
							8.60×10^{-1}		2.72×10^{-1}		
Tanikawa C, et al [7]	12q24.11-12	111335541	CUX2	rs6490061	C/T	0.682	1.08	(0.93 -1.25)	1.06	(0.96 -1.18)	0.808
							3.29×10^{-1}		2.45×10^{-1}		
Tanikawa C, et al [7]	20q11.21	31411284	DEFB121	rs2376549	C/T	0.283	1.14	(0.97 -1.33)	1.13	(1.01 -1.25)	0.844
							1.02×10^{-1}		2.91×10^{-2}		

OR, odds ratio; CI, confidence interval. [a] ORs were adjusted for age and sex.

Reference

1. Abnet, C.C.; Freedman, N.D.; Hu, N.; Wang, Z.; Yu, K.; Shu, X.O.; Yuan, J.M.; Zheng, W.; Dawsey, S.M.; Dong, L.M.; et al. A shared susceptibility locus in PLCE1 at 10q23 for gastric adenocarcinoma and esophageal squamous cell carcinoma. *Nat. Genet*. **2010**, *42*, 764–768, doi:10.1038/ng.649
2. Hu, N.; Wang, Z.; Song, X.; Wei, L.; Kim, B.S.; Freedman, N.D.; Baek, J.; Burdette, L.; Chang, J.; Chung, C.; et al. Genome-wide association study of gastric adenocarcinoma in Asia: A comparison of associations between cardia and non-cardia tumours. *Gut* **2016**, *65*, 1611–1618, doi:10.1136/gutjnl-2015-309340.
3. Jin, G.; Ma, H.; Wu, C.; Dai, J.; Zhang, R.; Shi, Y.; Lu, J.; Miao, X.; Wang, M.; Zhou, Y.; et al. Genetic variants at 6p21.1 and 7p15.3 are associated with risk of multiple cancers in han chinese. *Am. J. Hum. Genet*. **2012**, *91*, 928–934, doi:10.1016/j.ajhg.2012.09.009.
4. Sakamoto, H.; Yoshimura, K.; Saeki, N.; Katai, H.; Shimoda, T.; Matsuno, Y.; Saito, D.; Sugimura, H.; Tanioka, F.; Kato, S.; et al. Genetic variation in PSCA is associated with susceptibility to diffuse-type gastric cancer. *Nat. Genet*. **2008**, *40*, 730–740, doi:10.1038/ng.152.
5. Shi, Y.; Hu, Z.; Wu, C.; Dai, J.; Li, H.; Dong, J.; Wang, M.; Miao, X.; Zhou, Y.; Lu, F.; et al. A genome-wide association study identifies new susceptibility loci for non-cardia gastric cancer at 3q13.31 and 5p13.1. *Nat. Genet*. **2011**, *43*, 1215–1218, doi:10.1038/ng.978.
6. Wang, Z.; Dai, J.; Hu, N.; Miao, X.; Abnet, C.C.; Yang, M.; Freedman, N.D.; Chen, J.; Burdette, L.; Zhu, X.; et al. Identification of new susceptibility loci for gastric non-cardia adenocarcinoma: Pooled results from two Chinese genome-wide association studies. *Gut* **2017**, *66*, 581–587, doi:10.1136/gutjnl-2015-310612.
7. Tanikawa, C.; Kamatani, Y.; Toyoshima, O.; Sakamoto, H.; Ito, H.; Takahashi, A.; Momozawa, Y.; Hirata, M.; Fuse, N.; Takai-Igarashi, T.; et al. Genome-wide association study identifies gastric cancer susceptibility loci at 12q24.11-12 and 20q11.21. *Cancer Sci*. **2018**, *109*, 4015–4024, doi:10.1111/cas.13815.
